# Tissue and Serum miRNA Profile in Locally Advanced Breast Cancer (LABC) in Response to Neo-Adjuvant Chemotherapy (NAC) Treatment

**DOI:** 10.1371/journal.pone.0152032

**Published:** 2016-04-11

**Authors:** Manal Al-Khanbashi, Stefano Caramuta, Adil M. Alajmi, Ibrahim Al-Haddabi, Marwa Al-Riyami, Weng-Onn Lui, Mansour S. Al-Moundhri

**Affiliations:** 1 Medical Oncology Unit, Department of Medicine, College of Medicine and Health Sciences, Sultan Qaboos University, Muscat, Oman; 2 Department of Oncology-Pathology, Karolinska institute, Cancer Center Karolinska, Karolinska University Hospital-Solna, Stockholm, Sweden; 3 Department of Surgery, College of Medicine and Health Sciences, Sultan Qaboos University, Muscat, Oman; 4 Department of Pathology, College of Medicine, Sultan Qaboos University, Muscat, Oman; King Faisal Specialist Hospital &amp; Research center, SAUDI ARABIA

## Abstract

**Introduction:**

MicroRNAs (miRNAs) are small non-coding RNA that plays a vital role in cancer progression. Neo-adjuvant chemotherapy (NAC) has become the standard of care for locally advanced breast cancer. The aim of this study was to evaluate miRNA alterations during NAC using multiple samples of tissue and serum to correlate miRNA expression with clinico-pathological features and patient outcomes.

**Methods:**

Tissue and serum samples were collected from patients with locally advanced breast cancer undergoing NAC at four time points: time of diagnosis, after the first and fourth cycle of doxorubicin/cyclophosphamide treatment, and after the fourth cycle of docetaxel administration. First, we evaluated the miRNA expression profiles in tissue and correlated expression with clinico-pathological features. Then, a panel of four miRNAs (miR-451, miR-3200, miR-21, and miR-205) in serum samples was further validated using quantitative reverse-transcription polymerase chain reaction (RT-qPCR). The alterations in serum levels of miRNA, associations with clinical and pathological responses, correlation with clinico-pathological features, and survival outcomes were studied using Friedman, Mann-Whitney U, and Spearman, Wilcoxon signed-ranks tests. *P*≤0.05 was considered statistically significant.

**Results:**

We analyzed 72 tissue samples and 108 serum samples from 9 patients and 27 patients, respectively. MicroRNA expression profiling of tumor versus normal tissue revealed more than 100 differentially expressed miRNAs. Serum miR-451 levels were significantly decreased during treatment, and higher serum levels were associated with improved clinical and pathological responses and disease-free survival. This is one of the early reports on miR-3200 in response to treatment in breast cancer, as serum levels of miR-3200 found to decline during NAC, and higher serum levels were associated with lower residual breast cancer burden and relapse rates at time of diagnosis.

**Conclusion:**

Variations in serum miRNA levels during NAC treatment may be therapeutically significant for predicting response and survival outcomes.

## Introduction

Breast cancer is the most common cancer in females worldwide and therefore represents a significant global health burden[1]. The current treatment regimen of breast cancer involves multimodalities including chemotherapy, surgery, radiotherapy, hormonal treatment, and targeted therapy[2]. The choice and sequence of these modalities depend on the stage at presentation, estrogen receptor (ER) and progesterone receptor (PR), and human epidermal growth factor receptor 2 (Her2/*neu*) statuses. Currently, the administration of neo-adjuvant chemotherapy (NAC) prior to surgery is considered the standard practice in locally advanced breast cancer (LABC). NAC improves surgical resectability and breast conservation[3]. Moreover, several studies have suggested that patients who achieve a complete pathological response gain an overall survival benefit[4].

MicroRNAs (miRNAs) are a class of small non-coding RNA approximately 25nt in length that have unique functions at the post-transcriptional level [5, 6]. Mounting evidence from cancer research provides insights into the role of miRNAs in tumorigenesis [7, 8]. The first report on deregulation of miRNA in cancer was described in human lymphocytic leukemia [9], and subsequently, many other studies have revealed aberrations in miRNA expression in different cancer types, including breast cancer. Several studies have demonstrated differential miRNA-expression profiles in breast cancer patients compared with controls. A study by Iorio *et al*. identified a deregulated global pattern of 29 miRNAs when compared with normal breast tissues, strongly suggesting the importance of miRNA deregulation in breast cancer development [10]. Other studies have shown associations between miRNA expression and clinico-pathological features[11]. For example, expression of miR-30 is associated with ER and PR expression and miR-213 and miR-203 expression appears to be related to tumor stage. Similarly, Mattie *et al*. identified miRNA patterns in breast cancer associated with Her2/*neu* or ER/PR status[12]. Because of the stability of detectable miRNAs in whole blood, plasma, and serum in healthy controls and patients with breast cancer, circulating miRNAs also have been studied as a potential noninvasive biomarker to differentiate normal from cancerous states and to monitor responses to therapy. Moreover, it is also shown that some miRNA are capable of differentiating early stages of breast cancer from healthy controls [13]. A study comparing circulating miRNAs between patients with cancer and controls showed that circulating miR-195 levels in breast cancer decrease following tumor removal[14]. Two independent studies found elevated concentrations of circulating miR-21 in breast cancer patients versus control [15, 16]. Similarly, significantly higher levels of miR-29a and miR-598 were found in the serum of breast cancer patients. The circulating miR-10b, miR-34a, and miR-155 levels can discriminate between breast cancer patients and healthy individuals. Higher miR-155 circulating levels are found in PR-positive tumors compared with negative ones[17]. Wang *et al* showed that the relative expression of miR-21, miR-126, miR-155, miR-199a, and miR-335 is closely associated with breast cancer histologic tumor grade and sex hormone receptor expression status[18]. Circulating miR-21 differentiated patients with loco-regional disease from those with metastases[15]. Whereas some other studies showed distinctive miRNA expression patterns among different molecular subtypes [19]. Therefore, all above studies suggest the potential utility of miRNAs as predictive, diagnostic and prognostic biomarkers.

This current study focused on a subset of breast cancer patients with locally advanced disease, making it distinct from other studies that include all stages of breast cancer or primarily early stages. We focused on Locally Advanced Breast Cancer (LABC) cases for many reasons as it provides an excellent *in vivo* model to study the effect of NAC on miRNA profile as not many studies are available and also that many cases of the diagnosed patient in the Sultanate of Oman are of LABC nature. Moreover, this is the best *in vivo* clinical model where both tissue and serum miRNA expression alterations can be correlated with pathological response in particular which has been shown to predict survival.

We examined miRNA expression during NAC treatment in a dynamic fashion with multiple tissue and serum samples taken at various times over the course of treatment. The study hypothesized that miRNA dynamic expression levels in tissue and serum vary significantly during NAC at different time points. Moreover, these changes in miRNA expression during NAC correlate with clinical and pathological responses and survival.

## Materials and Methods

### Patients, treatment, and characterization

We recruited 27 consecutive patients diagnosed with locally advanced breast cancer undergoing NAC treatment at Sultan Qaboos University Hospital from 2010 to 2012. Patients were classified and staged according to the American Joint Committee on Cancer (AJCC) and tumor node metastasis (TNM) classification systems [20]. The NAC regimen included 4 cycles of doxorubicin hydrochloride (60mg/m^2^ intravenously [IV]) and cyclophosphamide (600mg/m^2^ IV) every three weeks, followed by 4 cycles of docetaxel (75mg/m^2^ [IV]) every three weeks. Patients with Her2/*neu* overexpression received trastuzumab (6mg/kg IV) with docetaxel once every three weeks for one year. The study was approved by the Ethical Committee at Sultan Qaboos University Ethics and was conducted in accordance to Helsinki Declaration. Written informed consent from each patient was obtained. The clinico-pathological data were retrieved from the Hospital Information System in accordance with the hospital’s privacy rules.

We evaluated the treatment responses of patients according to clinical and pathological outcomes. The clinical response was evaluated according to the World Health Organization (WHO) and the Response Evaluation Criteria In Solid Tumors (RECIST) 1.1 [21]:(1) complete response was defined as a complete disappearance of the tumor mass, (2) partial response was defined as a ≥50% reduction in the product of two perpendicular dimensions of the tumor mass, (3)progressive disease (PD) was defined as a ≥25% increase in the product of two perpendicular dimensions of tumor, and (4) stable disease (SD) was defined as a change that did not meet the criteria for any other categories. In this study, patients with complete and partial responses were categorized as responders, and patients with SD and PD as non-responders.

We determined the pathological response using the Residual Cancer Burden (RCB) index, which combines the pathological evaluation of tissue samples pre- and post-treatment, as described in Nahleh *et al* [22]. The RCB index is determined by measuring four important factors: (1) bi-dimensional widths of the primary tumor bed in the resection specimen (*d*_*1*_ and *d*_*2*_), (2) the proportion of the tumor bed encompassing invasive carcinoma (*f*_*in*_), (3) metastatic axillary lymph nodes (*LN*), and (4) the diameter of the largest metastasis in an axillary lymph node (*d*_*met*_). To calculate a single RCB class index, we calculated RCB_prim_ and RCB_met_ using the formula in which RCB_prim_ = *f*_*in*_*d*_*prim*_ and RCB_met_ = 4(1–0.75^*LN*^) *d*_*met*_. The RCB score was calculated using the MD Anderson Residual Cancer Burden Calculator (www3.mdanderson.org/app/medcalc/index.cfm?pagename=jsconvert3). We grouped patients with an RCB score of 0 or I as responders, and II or III as non-responders.

### Sample collection and storage

**Fig 1:**
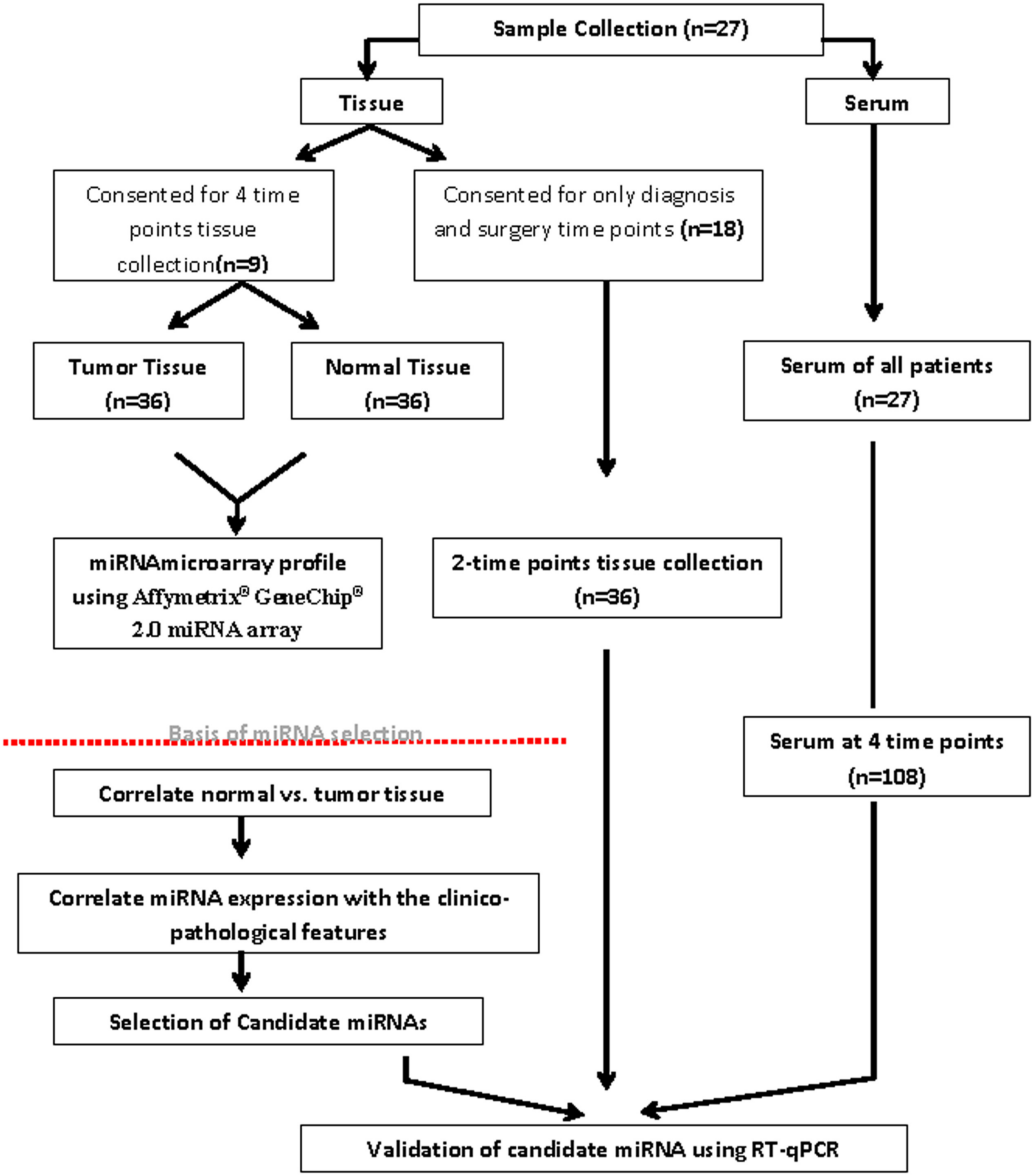
Schematic representation of the sample collection and study design.

From each patient (n = 9; out of the total 27 included in the study), matched tumor tissue (n = 36) paired with tumor-adjacent normal tissue (n = 36) and their peripheral blood samples (n = 36) were biopsied and collected by a breast surgeon using tru-cut biopsy needle gauge 14g x 10cm (BARD^®^ MC1410) at the time of diagnosis (baseline, point A), after the first cycle of doxorubicin/cyclophosphamide (point B), after the fourth cycle of doxorubicin/cyclophosphamide (point C), and at the time of surgery following 4 cycles of docetaxel treatment (point D). For the rest of the cohort (n = 18) few patients consented for biopsy tissue (normal and tumor) at diagnosis and surgical specimens, however peripheral blood samples were collected for all patients at 4 time points. Fig 1 summarizes the sample collection and study design (Fig 1). An Oncology nurse collected peripheral blood samples were collected from the patients attending for their chemotherapy treatment in the daycare unit and sera were obtained after they were allowed to coagulate at room temperature for at least 1hr and when the clot formation was observed it was centrifuged at 2000g for 10min. All samples were stored at -80°C until they were used for further analysis. Tissue samples were flash frozen with liquid nitrogen then stored at -80°C; serum was aliquoted and stored at -80°C. The samples did not undergo more than one freeze/thaw cycle before RNA extraction was performed.

### Tissue and serum total RNA isolation

Total RNA from tissue and serum (500μL) samples was extracted using the mirVana^TM^ miRNA isolation kit (Applied Biosystems/Ambion, Austin, TX, USA) in accordance with the manufacturer’s recommendation. Quantification of total RNA was performed using the Nanodrop (ND-1000, ThermoScientific, MA, USA)[23].

### MicroRNA microarray profiling

MicroRNA microarray profiling was performed on a cohort of 72 tissue samples from 9 patients at four different time points during treatment (36 tumor tissues and 36 tumor-adjacent healthy tissues) using the Affymetrix^®^ GeneChip^®^ 2.0 miRNA array (Affymetrix, Inc., Santa Clara, CA, USA). The microarray experiments were performed by EpigenDx, Inc. (Hopkinton, MA, USA) and the Bioinformatics and Expression Analysis core facility at Karolinska Institute, Sweden. A total of 500ng of RNA was labeled using the Flash Tag^TM^ Biotin HSR RNA labeling kit (Affymetrix, Inc., Santa Clara, CA, USA). Hybridization, washing, and staining were performed according to the standard protocols from Affymetrix. Arrays were scanned using the Affymetrix^®^ GeneChip^®^ Scanner 3000. We normalized the microarray data using the Cluster 3 software. The Significance Analysis of Microarrays (SAM) technique was used to identify significantly different expression of miRNA between healthy and tumor samples. The SAM data were then subjected to hierarchical clustering (http://statweb.stanford.edu/~tibs/SAM/) and correlation analyses with clinic-pathological features were performed and candidate miRNA were subsequently validated.

### Quantitative reverse-transcription polymerase chain reaction (RT-qPCR)

We determined the expression levels of miR-21, miR-3200, miR-451, and miR-205 using the predesigned TaqMan MicroRNA assay (Life Technologies, Foster City, CA, USA). We synthesized cDNA from 100ng of total RNA using the TaqMan^®^ MicroRNA Reverse Transcription kit (Life Technologies) and performed RT-qPCR using the 7900HT Fast Real-Time PCR System (Life Technologies)[24]. RNU6B or miR-191[25] was used as an endogenous control to normalize tissue or serum samples[26], respectively. All reactions were performed in triplicate, and relative expression of miRNA throughout the treatment time points was determined using the C_T_ method and reported as 2^-ΔCT^.

### Statistical analysis

Univariate analysis of the Friedman test was used to assess changes in miRNA expression across different time points. At each time point, the Mann-Whitney U test was applied to assess significant changes in the expression levels of miRNA in relation to treatment response and based on RCB class. Bivariate Spearman's rank correlation coefficient analysis was performed for all correlation analyses. Pair-wise comparisons of mina expression at points A (baseline) and D (end of treatment) were evaluated using the Wilcoxon signed-ranks test. Disease-free survival (DFS) was measured from the date of diagnosis until patient relapse and/or censor at the date of last follow up. Overall survival (OS) was measured from the date of diagnosis to the date of death or date of analyses. Both OS and DFS analyses were evaluated using Kaplan-Meier curves and the log-rank test. Statistical analyses were performed using SPSS v.21.0 Software (SPSS Inc., Chicago, IL), and *P*≤0.05 was considered statistically significant.

## Results

This study enrolled 27 patients with locally advanced breast cancer attending the Sultan Qaboos University Hospital from 2010 to 2012. The clinico-pathological features are summarized in Table 1. The cohort of patients was relatively young compared with other studies, with a mean age (±SD) of 43.33 years (±10.07). We found significant breast cancer burden in these patients; clinical T3 and T4 lesions were present in 77.8% of patients, and N2/N3 lymph node and skin involvement were found in 41.7% and 44.4% of patients, respectively, as shown in Tables 1 and 2. Luminal (A+B), triple-negative, and Her2/*neu* subtypes were found in 77.8%, 14.8%, and 7.4% of patients, respectively.

**Table 1:** Clinical characteristics of enrolled patients before chemotherapy (n = 27).

Characteristics	n	%
**Age**		
**≤45**	15	55.6
**>45**	12	44.4
**Menopausal status**		
**Pre-menopausal**	17	62.96
**Post-menopausal**	10	37.04
**Skin involvement**		
**Yes**	12	44.4
**No**	15	55.6
**Tumor Differentiation**		
**Well and Moderate**	14	51.9
**Poor**	13	48.1
**Estrogen receptor status**		
**ER+**	21	77.8
**ER-**	6	22.2
**Progesterone receptor status**		
**PR+**	19	70.4
**PR-**	8	29.6
**Her2/neu receptor status**		
**Her2/*neu*+**	7	25.9
**Her2/*neu*-**	20	74.1

**Table 2:** Cancer staging, clinical responses, and pathological responses before and after treatment.

Cancer staging, clinical and pathological responses					
Before Chemotherapy			After Chemotherapy		
Characteristics	n	%	Characteristics	n	%
**T stage (T)**			**T stage(T)**		
**T0**	0	0	**ypT0**	2	7.4
**T1**	4	14.8	**ypT1**	8	29.6
**T2**	2	7.4	**ypT2**	2	7.4
**T3**	4	14.8	**ypT3**	5	18.5
**T4**	17	63.0	**ypT4**	10	37.0
**LN stage(N)**			**LN stage(N)**		
**N0**	7	25.9	**ypN0**	9	33.3
**N1**	8	29.6	**ypN1**	9	33.3
**N2**	8	29.6	**ypN2**	5	18.5
**N3**	4	14.8	**ypN3**	4	14.8
**Metastasis(M)**			**Metastasis (M)**		
**Absent**	23	85.2	** Absent**	23	85.2
**Present**	4	14.8	** Present**	4	14.8
**TNM Clinical stage**			**TNM Pathological stage**		
**I**	0	0	**I**	4	14.8
**II**	9	33.3	**II**	10	37.0
**III**	14	51.9	**III**	9	33.3
**IV**	4	14.8	**IV**	4	14.8
**Overall Clinical Response**			**Residual Cancer Burden (RCB)**		
**cCR**	5	18.5	**0**	0	0
**cPR**	18	66.7	**I**	4	14.8
**cSD**	4	14.8	**II**	14	51.9
**cPD**	0	0	**III**	9	33.3

CS = clinical stage, T = clinical tumor stage, N = clinical lymph node stage, M = metastasis, ypT+ ypN = pathological stage after treatment of tumor and lymph node, PS = pathological stage after treatment.

### Clinical and pathological responses

The clinical and pathological responses are presented in Table 2. The clinical response assessments showed that, after chemotherapy, 5 patients (18.5%) achieved a complete clinical response and 18 patients (66.7%) achieved a partial response with no progressive disease. The pathological responses included a reduction in T stage after chemotherapy with a decrease in the percentage of patients with T4 lesions (from 63% to 37%) and an increase in the percentage of patients with T1 lesions (from 14.8% to 29.6%). However, only 2 patients (7.4%) achieved a complete pathological response of the primary tumor lesions and lymph nodes, as shown in Table 2. We further assessed pathological response by calculating the RCB score, which takes into account the response in the primary tumor mass and lymph nodes. The RCB scores were0, II, III, and I in 0, 4, 14, and 9 patients, respectively. We did not detect any changes in ER/PR status or Her2/*neu* status after chemotherapy.

### Differential expression of miRNAs in tumor versus tumor-adjacent healthy tissue

**Fig 2:**
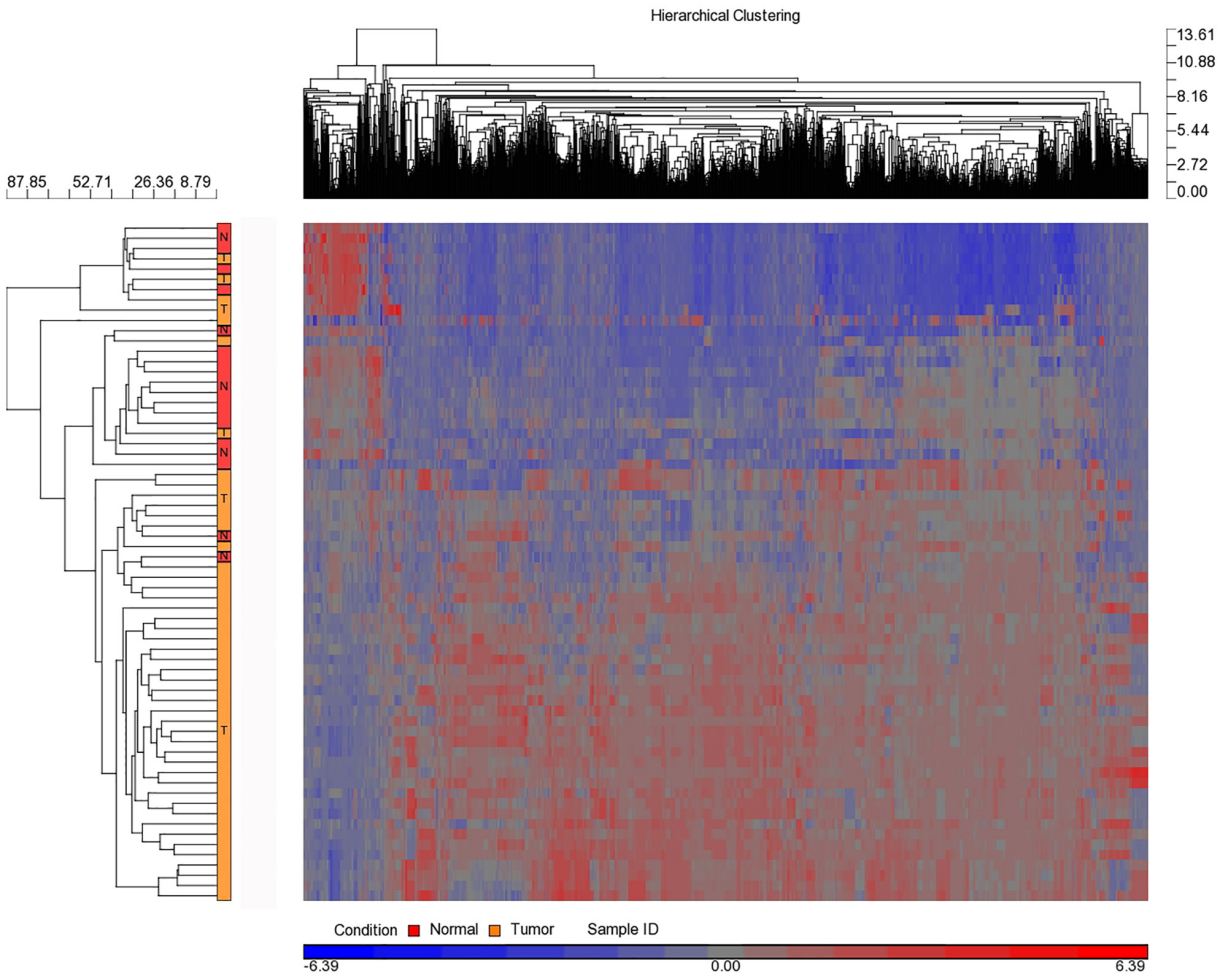
Hierarchal clustering of healthy versus tumor breast cancer tissue samples. Over 100 detected miRNAs that were differentially expressed. Heat map colors represent miRNA expression as indicated in the color key where tumor samples are from the all patients whereas normal tissue from the full follow-up patients.

**Fig 3:**
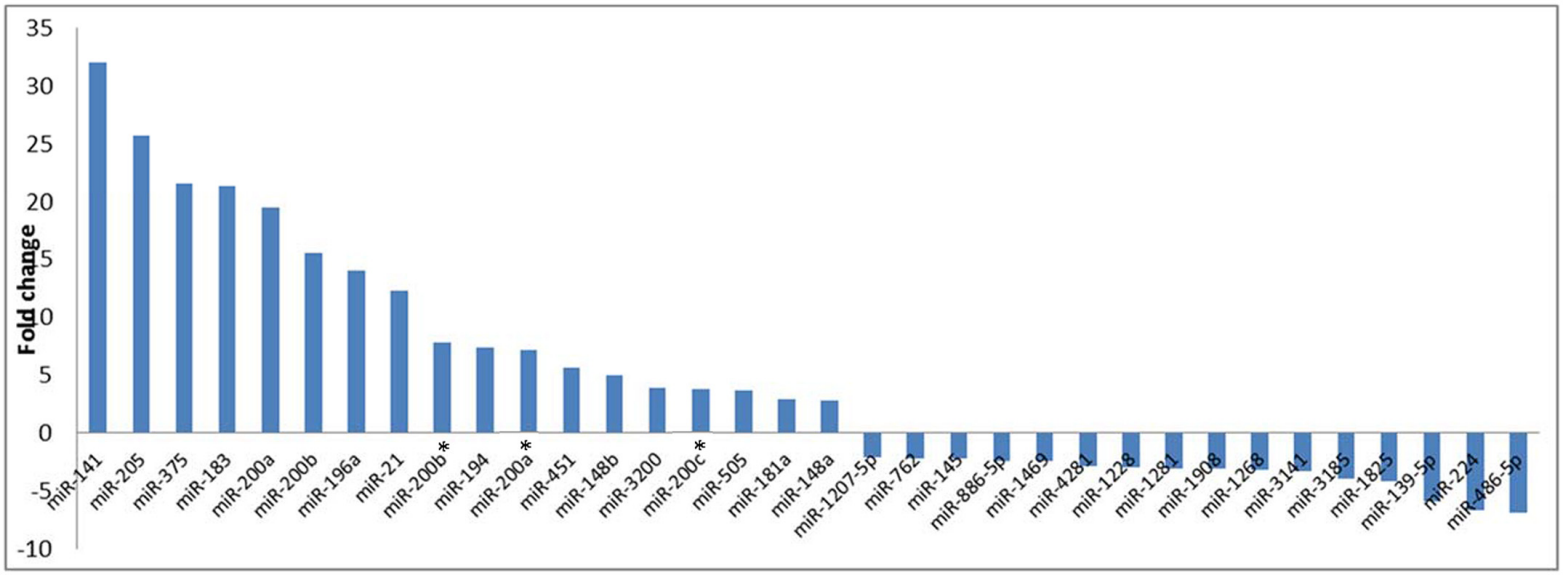
Differential expression of some randomly selected miRNAs between normal and breast cancer tumor tissue samples with various fold changes.

MicroRNA expression profiling of normal tissue (n = 9) versus tumor (n = 9) at four time points (total 72 samples of the 4 follow-ups and the normal of two time points A and D) revealed more than 100 differentially expressed human miRNAs (Fig 2 and S1 Table). Fig 3 presents some of the miRNAs that significantly differed in expression level (*P*<0.05) prior to neo-adjuvant chemotherapy administration. Some miRNA levels were increased 2- to 32-fold, whereas other miRNA levels were under-expressed in cancer tissues 2- to 6-fold.

### The correlation between tissue miRNA levels and clinico-pathological features

**Fig 4:**
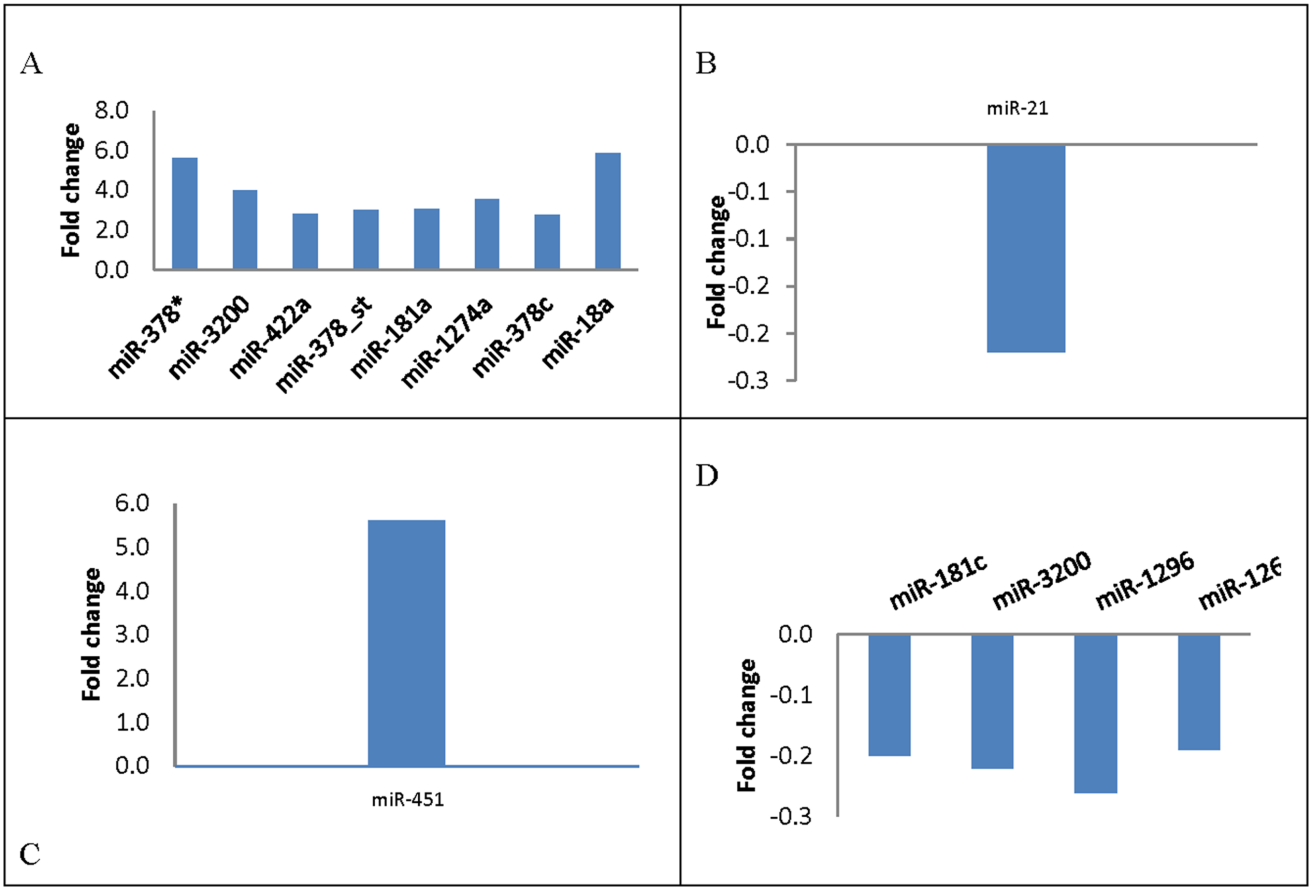
Differential expression of miRNAs in relation to clinico-pathological features using SAM analysis. (A) Tissue miRNAs significantly associated with Estrogen receptor (ER+) expression (*p*≤0.05). (B) Tissue miRNAs significantly associated with relapse (*p*≤0.05). (C) Tissue miRNAs significantly associated with pathological stage (*p*≤0.05). (D) Tissue miRNAs significantly associated with Basal-like (triple negative) (*p*≤0.05).

The statistical technique SAM was used to identify which specific miRNA expression correlated with clinico-pathological features at base line level. With false-discovery rate of no more than 0%, the analysis identified 8 deregulated miRNAs correlated with ER-positive patients, 1 miRNA correlated with recurrence, 1 miRNA correlated with pathological stage, and 4 miRNAs correlated with triple-negative breast cancer (Fig 4).

Because of the large number of differentially expressed miRNAs, we selected only few miRNAs for further validation in serum. These were chosen based on their significant differential expression between tumor tissue and tumor-adjacent normal tissue (>2-fold increase) and their correlation with clinico-pathological features either strong positive or negative. Therefore, miR-21, miR-3200, miR-451, and miR-205 were selected for further validation in tissue and serum. For these miRNAs, we analyzed their expression levels over the course of the NAC treatment as well as their correlation with clinico-pathological features, clinical and pathological responses, and survival outcomes.

### Tissue miRNA levels alteration during NAC treatment

Tissue levels of miR-451, miR-3200, miR-21, and miR-205 were validated at various times during NAC. We found no statistically significant differences in fold change of miRNA levels between points A and D in the normal tissue tumor-adjacent (n = 5). Moreover, we found no statistically significant differences in miRNA levels alterations in the tumor tissue between points A and D (n = 10) and at all-time points where full set of tumor tissue samples were available for patients (n = 8).

### Tissue versus serum miRNA level during NAC treatment

We correlated tissue and serum miRNA levels at various time points. We found that at the abundance of the miRNAs was highly variable between tumor tissue and serum samples collected prior to administration of chemotherapy. They were all more highly expressed in tissue than serum, except for miR-451, whose expression was 19 times higher in serum compared with tumor tissue. During NAC treatment, the tissue and serum miRNA levels did not correlate at any time point.

### Changes in serum miRNA levels during NAC treatment

The serum levels of miR-451, miR-3200, miR-21, and miR-205 were studied at four time points and had varied expression patterns among patients; for instance, miR-451 relative expression ranged from 0.00 to 478.14 with median of 46.93.

**Fig 5:**
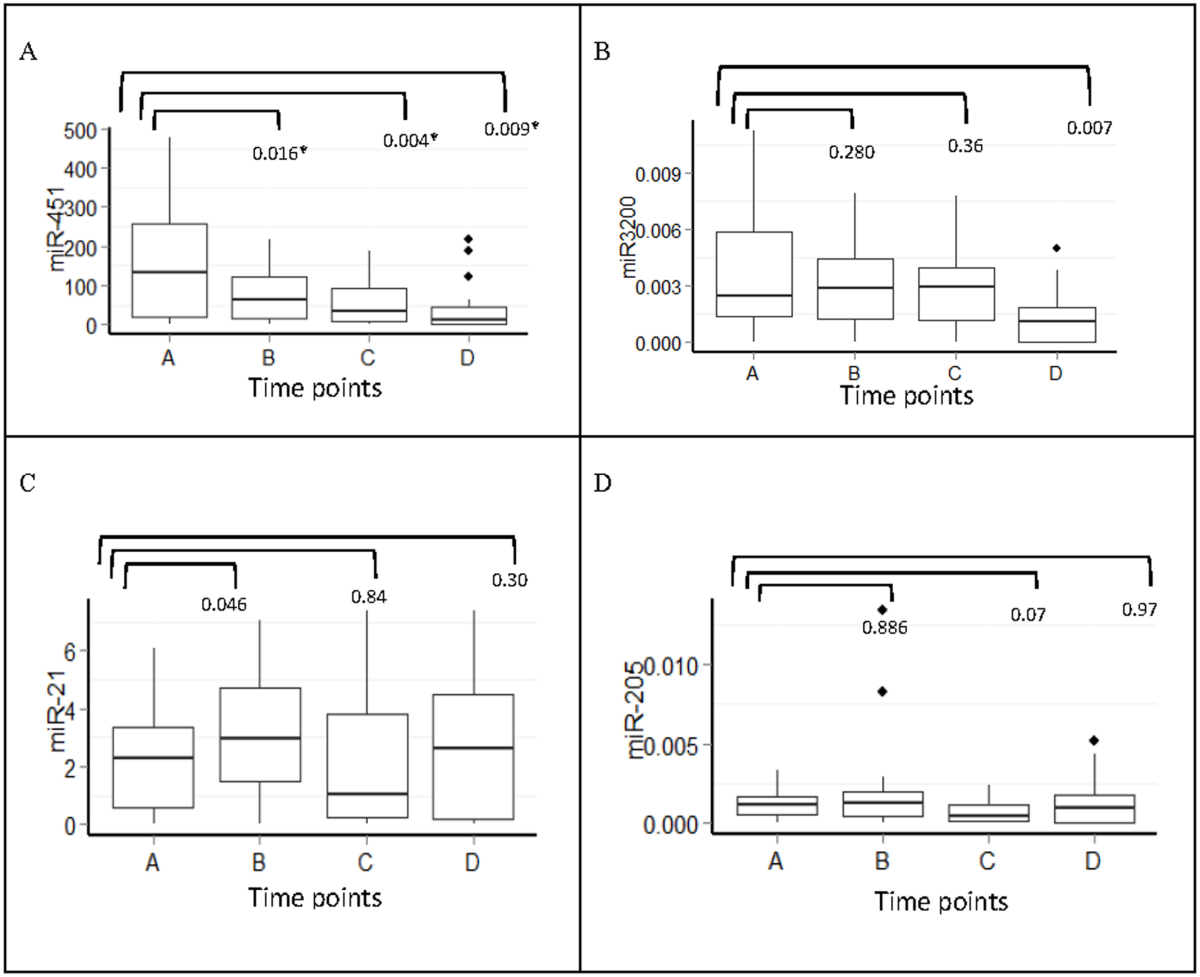
Serum miRNAs fold change expression at different time points during chemotherapy treatment (n = 27). A) miR-451, B) miR-3200, C) miR-21and D) miR-205, * Significant p values of *p*<0.05 Mann-Whitney U test. Y-axis represents the median expression of 2^-ΔCT^ value of each miRNA.

MiR-451 showed significant changes with treatment (*P* = 0.032, Friedman test); furthermore, certain time points were significantly different as assessed by pair-wise comparison. Interestingly, we found a gradual decline in serum levels of miR-451 during doxorubicin/cyclophosphamide (points B and C) and taxane treatment (point D). A significant decline in serum levels of miR-3200 was only observed following taxane treatment (point D) (*P* = 0.007). By contrast, there was an increase in miR-21 serum levels at point B (*P* = 0.046) and a non-significant increase at point D (*P* = 0.300). MiR-205 showed no significant variation, likely because of its very low concentration in serum (Fig 5).

### Correlation between serum miRNAs and clinico-pathological features

The correlation between serum miRNA levels and clinico-pathological features at various time points is shown in Table 3. The pre-chemotherapy miR-3200 serum levels were inversely correlated with lymph node clinical stage (r = -0.44, *P* = 0.02). Similarly at baseline, miR-21 expression correlated inversely with ER status (r = -.49, *P* = 0.01). After the first dose of doxorubicin/cyclophosphamide treatment, Her2/*neu* status correlated with miR-3200 and miR-205 expression (r = 0.48, P = 0.01; r = 0.46, *P* = 0.02, respectively). At point C (the end of doxorubicin/cyclophosphamide treatment), ER status correlated inversely with miR-451, miR-3200, miR-21, and miR-205 expression (r = -0.52, -0.49, -0.59, -0.45; *P* = 0.01, 0.009, 0.003, 0.03, respectively).

**Table 3:** Bivariate Spearman’s rank correlation coefficient of miRNA expression and clinico-pathological characteristics (n = 27).

Time points	miRNA	G	CS	T	N	M	ypT	ypN	CS	PS	SI	ER	PR	Her2/*neu*
**A**	miR-451	-.021	.119	.133	-.076	.057	-.226	-.129	-.126	.038	.195	-.085	.178	.306
	miR-3200	.305	-.038	.074	-.441*	-.254	-.356	-.260	-.176	-.180	-.297	-.298	-.063	-.217
	miR-21	.262	.034	-.114	-.079	-.147	-.196	-.351	-.219	-.050	-.048	-.492**	-.260	-.011
	miR-205	.012	-.083	-.343	-.365	.291	-.228	.141	-.089	-.321	-.145	-.081	-.042	.265
**B**	miR-451	.190	.343	.172	-.045	.257	.102	-.244	.245	.190	.235	-.250	-.111	.325
	miR-3200	.247	.165	-.245	.141	.107	-.351	-.175	-.171	-.367	.115	-.217	-.229	.478*
	miR-21	.067	.239	-.037	.046	.134	-.139	-.367	.258	.150	.258	-.297	-.135	.293
	miR-205	-.062	.179	-.083	.274	-.056	-.288	-.261	.070	-.113	.296	-.013	-.058	.457*
**C**	miR-451	.354	.107	.097	-.377	.048	-.297	-.177	-.240	-.094	-.157	-.517**	-.445*	-.258
	miR-3200	.411*	.025	-.420*	-.052	-.102	-.235	-.009	-.289	-.348	-.067	-.485*	-.284	-.039
	miR-21	.260	.093	.011	-.172	.097	-.156	-.317	-.054	.099	-.157	-.585**	-.298	-.192
	miR-205	.053	-.038	.031	.022	-.195	-.115	-.377	.154	.100	-.151	-.450*	-.112	-.036
**D**	miR-451	.150	-.119	.164	-.629**	.067	.046	-.096	.088	-.091	-.480*	.166	.467*	-.104
	miR-3200	-.280	-.287	-.088	-.377	-.086	-.263	-.047	-.232	-.681**	-.431	.455	.455	-.191
	miR-21	.105	-.039	.075	-.332	.121	-.130	-.101	-.054	-.122	-.386	.078	.345	.086
	miR-205	.151	.305	-.026	-.029	.052	-.044	-.135	-.076	-.201	-.168	-.056	.258	.223

G = tumor grade (differentiation), CS = clinical stage, T = clinical tumor stage, N = clinical lymph node stage, M = metastasis, ypT+ ypN = pathological stage after treatment of tumor and lymph node, PS = pathological stage after treatment, SI = skin involvement, ER = estrogen receptor, PR = progesterone receptor.

Spearman correlation*P*-values:

* *P*<0.05 (2-tailed),

** *P*<0.01 (2-tailed).

Moreover miR-3200 expression correlated positively with tumor grade (differentiation) (r = 0.4, *P* = 0.04), but inversely with tumor clinical stage (r = -0.4, *P* = 0.03). At point D (the end of docetaxel treatment), miR-3200 expression correlated inversely with pathological stage and miR-451 expression inversely correlated with clinical lymph node involvement and skin involvement.

**Fig 6:**
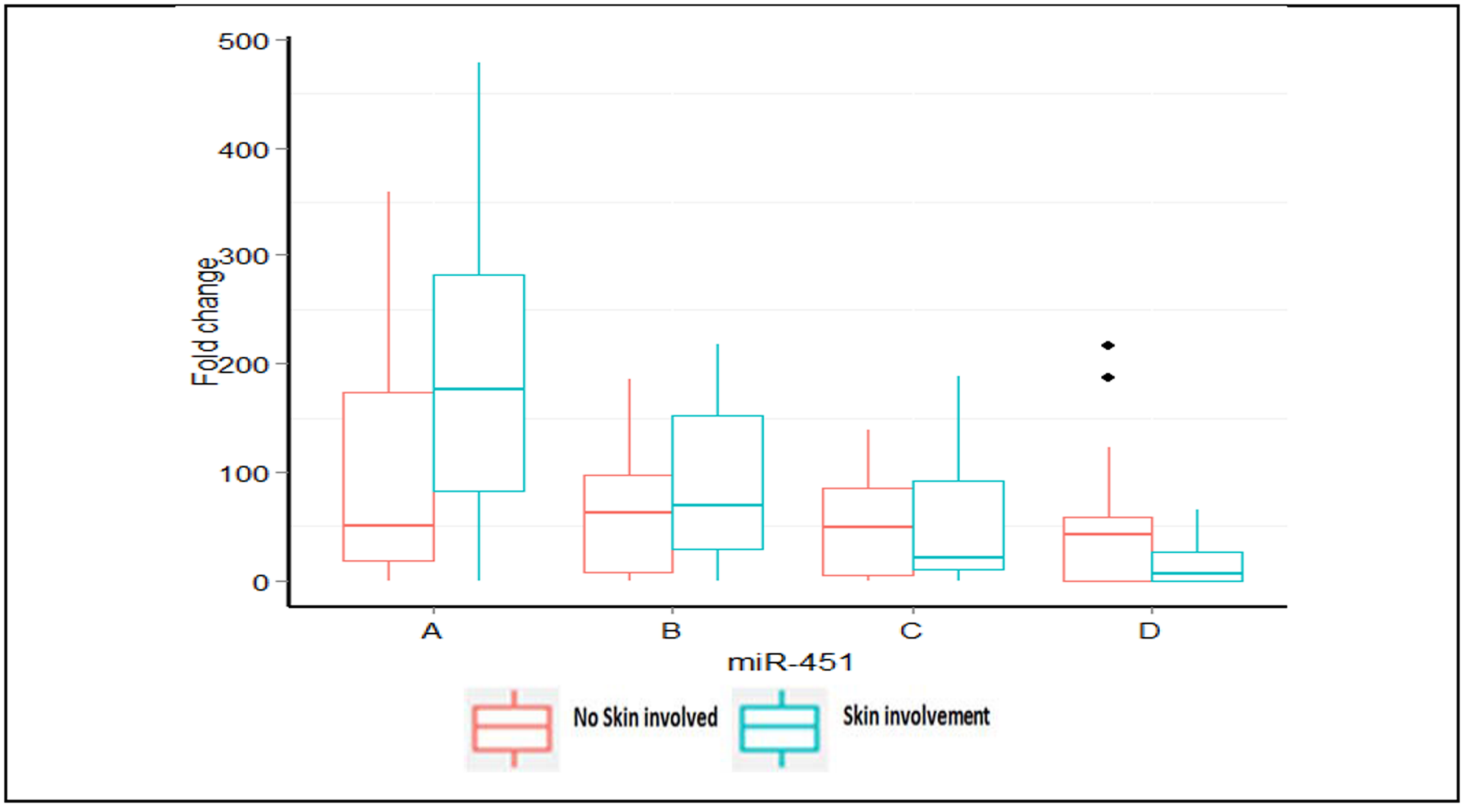
Correlation between miRNA expression and skin involvement. Boxplot of median miR-451 expression comparing patients with skin involvement versus patients with no skin involvement at different time points throughout chemotherapy (n = 27), Spearman correlation (*r* = -0.48, *P* = 0.028).

In patients with skin involvement, miR-451 expression decreased substantially with treatment, approximately 20 fold from the initial level at time of diagnosis until the end of treatment (*P* = 0.003; Fig 6). Spearman correlation analysis consistently showed that patients with skin involvement exhibited a significant reduction in miR-451 expression at the end of treatment (point D)compared with those that had no skin involvement (r = -0.48, *P* = 0.028).

### The association between serum miRNA levels and clinical and pathological responses

**Fig 7:**
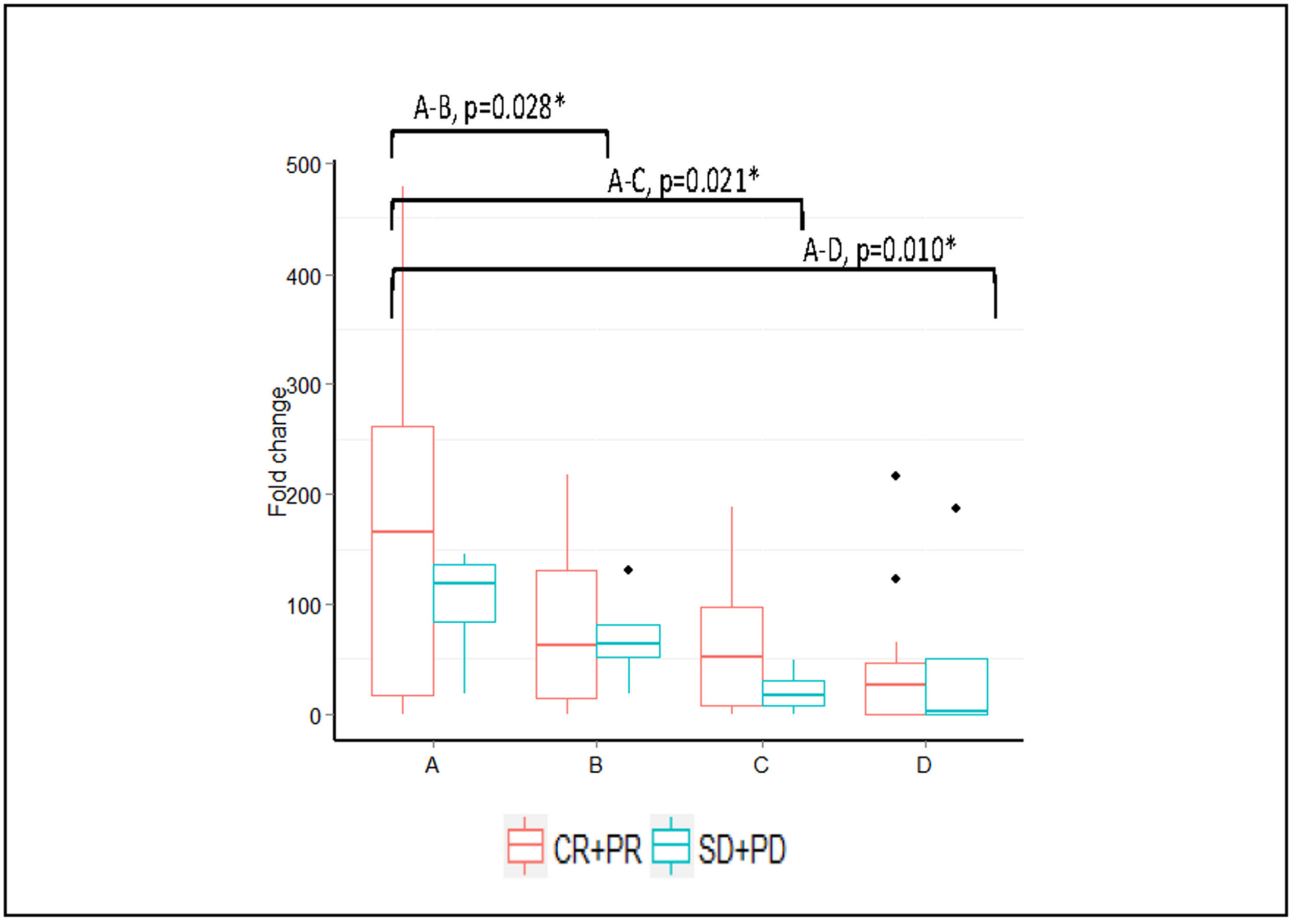
Median fold change expression changes in miR-451 in clinical responders (data shown for the clinical responders only). *Significant *p* values of *p*<0.05 Wilcoxon sign rank test (n = 27).

When patients were stratified as clinical responders (complete and partial responses) and non-responders (SD+PD), only miR-451 showed overall significant changes (*P* = 0.038) in the clinical responders, with changes seen at points A-B, A-C, and A-D (*P* = 0.028, 0.021, and 0.010, respectively). The non-responders showed no significant overall changes at any time point (*P* = 0.494; Fig 7).

**Fig 8:**
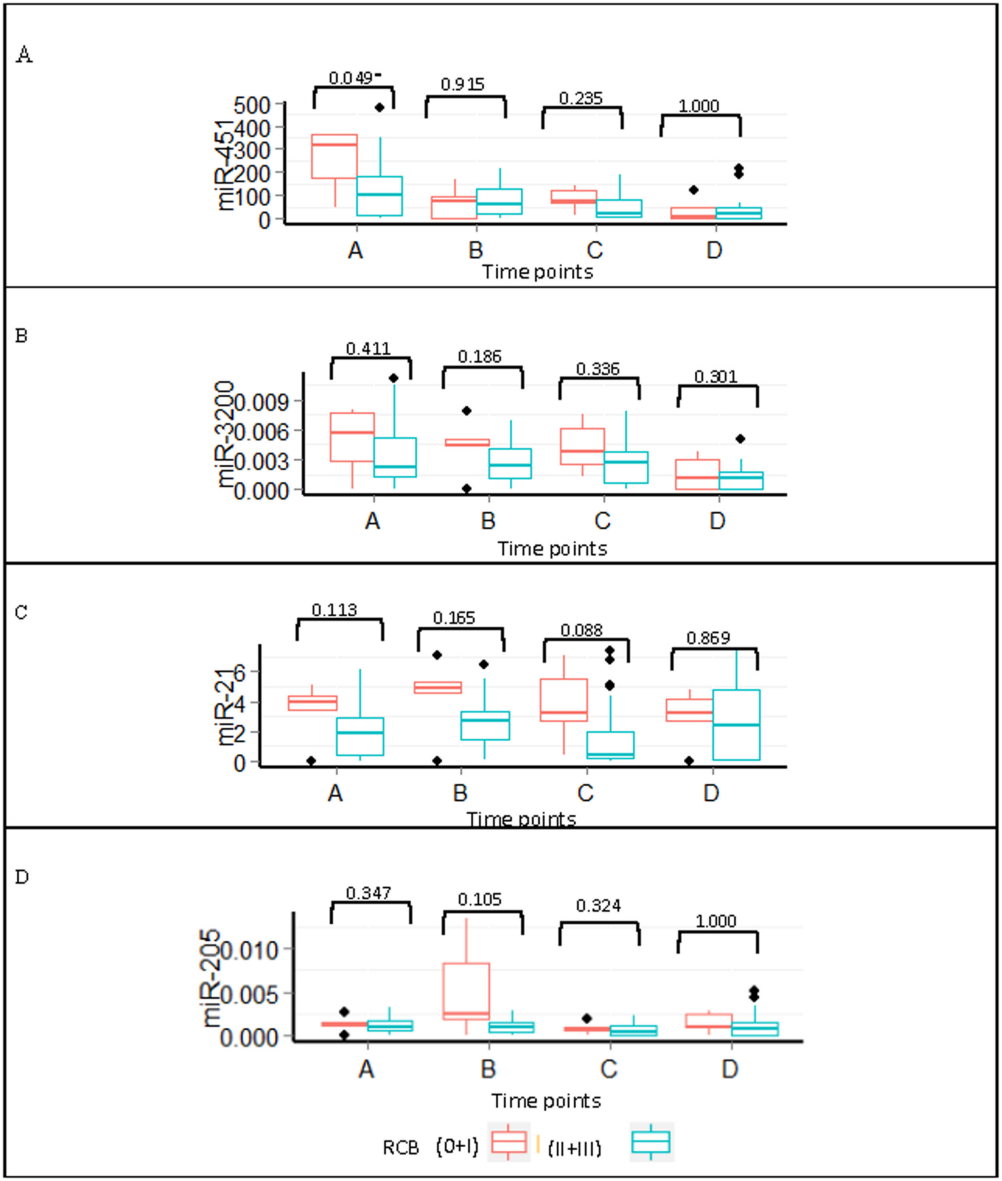
Expression of miRNA throughout treatment for pathological responders and non-responders. Boxplots of the (A) miR-451, (B) miR-3200, (C) miR-21, and (D) miR-205 expression during treatment for patients with a pathological response (0+I) versus no response (II+III). * P<0.05 using the Mann-Whitney U test.

Patients also were stratified according to their RCB status into responders (n = 5) and non-responders (n = 22). We found no statistically significant differences between responders and non-responders in the serum levels of miR-451, miR-3200, miR-21, or miR-205 during chemotherapy treatment. However, miR-451 at baseline levels was higher in responders versus non-responders (*P* = 0.049). The expression levels of all miRNAs in the responders remained elevated throughout the treatment (Fig 8).

### Survival analysis

**Fig 9:**
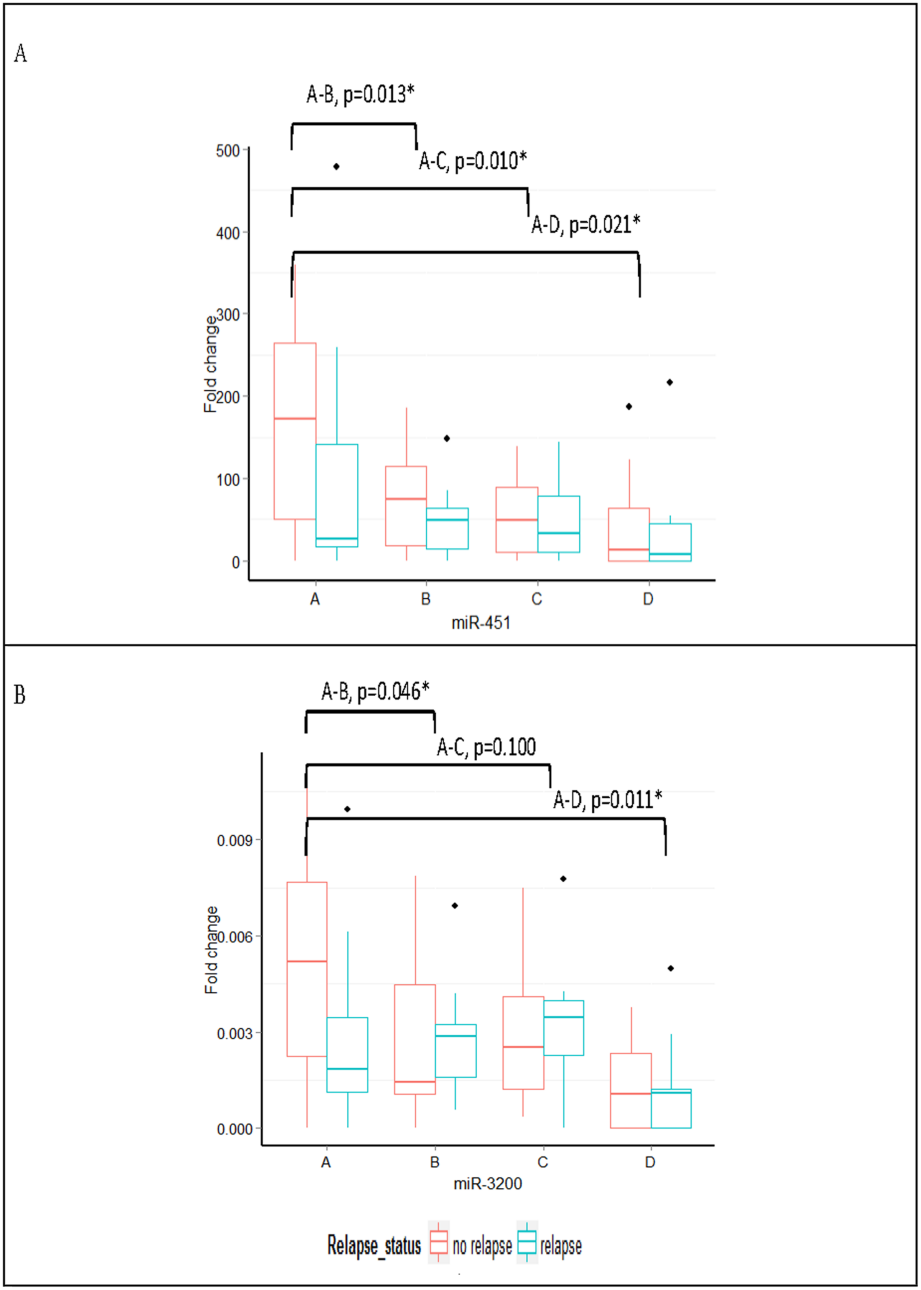
Fold change in miR-451 and miR-3200 expression during treatment in relation to relapse status. Boxplots of (A) miR-451 and (B) miR-3200 expression over time comparing relapsed patients versus non-relapsed. *P<0.05 using the Wilcoxon sign-rank test for the non-relapsing patients.

By the date of the survival analysis, 11 patients had relapsed and 6 patients had died. The median DFS was 39 months (13–51 months) with a median OS of 41 months (20–52 months). Levels of miR-451 were significantly different with treatment between the patients that did not relapse (n = 10; *P* = 0.033). However such alterations miR-451 levels were not observed in those who relapsed (n = 11; *P* = 0.392). The levels ofmiR-3200 showed a similar pattern tomiR-451 alterations in patients who continued to be disease versus those who relapsed (*P* = 0.022 and *P* = 0.532, respectively)(Fig 9). The levels of miR-21 and miR-205 showed no significant changes throughout the treatment.

**Fig 10:**
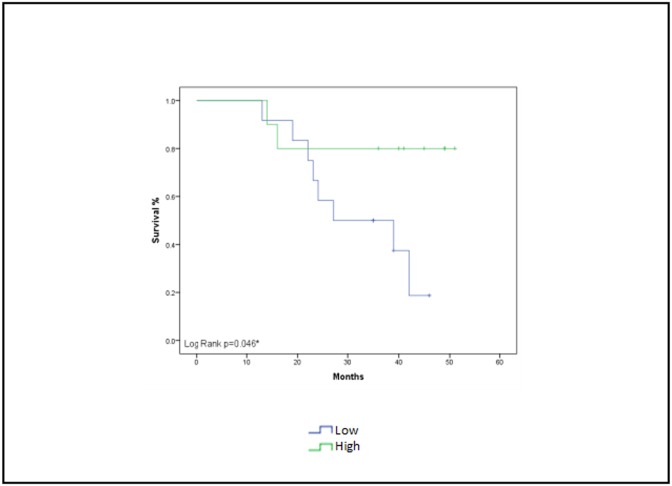
Disease-free survival and miR-451 expression. Kaplan-Meier estimates of disease-free survival based on low versus high expression of miR-451 at the time of diagnosis. Log-rank *P* = 0.046.

The serum levels of miR-3200, miR-21, and miR-205 at any point during chemotherapy treatment were not predictive for DFS; however, high levels of miR-451 at the time of diagnosis were associated with better DFS than were low levels (43 months [14–51 months] versus 31 months [13–46 months], respectively; Fig 10). None of the miRNAs was predictive for OS.

## Discussion

The current study investigated the effect of NAC treatment (doxorubicin/cyclophosphamide x4 followed by docetaxel×4 ±trastuzumab) on miRNA expression in subsets of patients with locally advanced breast cancer. We used a unique, dynamic method in which multiple tissues and serum samples were collected at four time points; the changes in miRNA levels over time were correlated with clinico-pathological features, clinical and pathological response, and survival.

In this study, we demonstrated differential over- and under-expression of more than 100 miRNAs between tumor and tumor-adjacent healthy tissues with fold change that varied from -6.90 to +31.99 (Figs 2 and 3). Several previous studies have demonstrated differential expression of miRNAs between various types of cancer and the adjacent healthy tissue [27, 28].

Tissue miRNA levels were associated with various clinico-pathological features (Fig 4). Tissue miR-378, miR-3200, miR-422a, miR-181, miR-1274a, miR-378c, and miR-18a were significantly associated with ER expression (*P*≤0.05). Specifically, we observed a 6-fold increase in the differential expression of miR-378 and miR-181. Previous studies have shown that miR-378 promotes cell survival by decreasing caspase-3 activity and stimulates tumor progression and ER positivity, which are consistent with our findings [29]. Previous studies have also found a tumor-suppressive role of miR-422a and its up-regulation in ER-positive inflammatory breast cancer, which is also consistent with our findings (Fig 4)[30, 31]. Tissue miR-21 was previously shown to be significantly associated with tumor local and distant recurrence and exhibits an oncogenic function in which its suppression results in enhanced sensitivity to anticancer agents[32]. Similarly, It was shown that Serum miR-21 may be an independent poor prognostic factor for recurrence [32], and high stromal miR-21 expression is associated with significantly shorter recurrence-free survival in patients with colorectal cancer [33]. We found that tissue miR-451 was up-regulated more than 5× and significantly associated with pathological stage(Fig 3). Previous studies have demonstrated that miR-451 regulates the expression of multidrug resistance 1 gene, and transfection of MCF-7/DOX-resistant cells with miR-451 results in increased sensitivity to chemotherapy [34].

Interestingly, down-regulation of tissue miR-181, miR-3200, miR-1296, and miR-1269 is associated with triple-negative breast cancer, but we cannot make a definitive assertion about the differential expression of these miRNAs in our cohort because only a few patients with this subtype were enrolled in our study. Scarce data are available about some of these miRNAs, for example, miR-181 and miR-1296 is significantly down-regulated in prostate cancer samples [35]; miR-1269 expression is up-regulated in human hepatocellular carcinoma and its inhibition reduces proliferation, tumorigenicity, and cell cycle progression [36]. However, it has been also reported that miR-181a and b were overexpressed in triple-negative breast cancer cases [37].

We performed RT-qPCR on four miRNAs (miR-451, miR-3200, miR-21, and miR-205) in the serum of 27 patients as these miRNA were selected based on their correlation to patients’ clinico-pathological features. The miRNAs were chosen because they had the highest differential expression and/or correlated with some of the clinico-pathological features, as discussed above.

We first studied whether serum miRNA expression varied significantly during NAC. Interestingly, we found a gradual decline in serum levels of miR-451 during doxorubicin/cyclophosphamide treatment (point B [*P* = 0.016] and point C [*P* = 0.004]) that continued during taxane treatment (point D [*P* = 0.009]; Fig 5). Similar studies reported a correlation between miR-451 decline and chemosensitivity in response to doxorubicin treatment in MCF-7 cells [38]. By contrast, we saw a significant decline in serum levels of miR-3200 only after taxane treatment (point D [*P* = 0.007]). We found no significant variation in miR-21 serum levels, except a minor statistically non-significant increases in expression at point B (*P* = 0.05) and point D (*P* = 0.3). We also noted no significant variation in miR-205, most likely owing to its very low serum concentration. Taken together, these data suggest that miRNA expression varies during neo-adjuvant treatment, which could be taken into consideration for the utilization of miRNAs as a biomarker [39–41].

Expression of miRNA was correlated with certain clinico-pathological features of enrolled patients. At the end of the treatment, miR-451 correlated inversely with clinical lymph node stage (*P* = 0.002) and skin involvement (*P* = 0.028). Patients with skin involvement had high levels of miR-451at point A that were subsequently down-regulated, whereas patients without skin involvement had almost steady levels (Fig 6). Previous studies have shown that the down-regulation of miR-451 expression in non-small cells lung carcinoma (NSCLC) correlates with advanced stage of disease, including differentiation and lymph node stage [34]. We found that miR-3200 correlated inversely with clinical lymph node stage (N) and clinical tumor stage (T) by the end of the doxorubicin/cyclophosphamide treatment (point C). However, we found no previous studies on its role in carcinogenesis. Expression of miR-21 correlated inversely with ER status after the administration of doxorubicin/cyclophosphamide (point C), which was lost by end of taxane treatment. Studies show that activation of ER in MCF-7 cells by estradiol represses the oncogenic effect of miR-21 [42]. These findings suggest that correlation with a fixed time point during neo-adjuvant chemotherapy treatment may provide inaccurate snapshot that does not take into account the dynamic alteration of various miRNAs.

We also correlated the clinical and pathological responses with miRNA expression. We found a gradual decline in miR-451 expression in the clinically responding patients versus non-responding patients. Interestingly, higher pre-treatment serum levels of miR-451 were associated with better clinical responses, as shown in Fig 6. This observation is consistent with a previous study in osteosarcoma that reported a correlation between high expression of serum miR-451 and positive responses to NAC, suggesting that miR-451 can be used as a pivotal marker to predict response to chemotherapy [43]. The other miRNA did not exhibit any significant differences between the clinically responding patients versus non-responding patients.

The pattern of miR-451 expression was retained in the pathological responding patients versus non-responding patients with gradual declined near the end of the treatment. The other miRNA remained unchanged when categorized by pathological response (Fig 8). Despite the use of standard chemotherapy treatment, the number of patients achieving complete pathological tumor response was low (7.4%) when compared with previous studies. This may reflect the very advanced nature of breast cancer lesions included in the study, with an average size of 8cm (SD = ±5cm), stage III and IV disease constituting 66.7% of cases, and skin involvement in approximately 44% of patients. Another characteristic that may have affected pathological response is the predominance of ER/PR-positive tumors and low number of patients with the triple-negative phenotype.

The findings presented in Fig 9 show that patients with higher miR-451 levels at baseline have lower relapse rates and better survival. This is supported by multiple studies that suggest miR-451 is tumor suppressive. Studies also have shown that low expression of miR-451 is an independent factor associated with worse OS for patients with NSCLC [34]. Expression of miR-451 is associated with clinical outcome in several cancers, including gastric and lung cancer. Specifically, patients with gastric cancer who had low miR-451 expression had a shorter DFS and OS than patients with high miR-451 expression, which is consistent with our study (Fig 10) [34]. However, it is important to highlight the relatively higher concentration of serum of miRNA-451 compared to tissue concentration which obviously raises the issue of exogenous sources of miRNAs.

Expression of miR-3200 has not been studied in cancer and very little is known about its function and mechanism of action. In the current study, we demonstrated that serum levels of miR-3200 declined throughout the chemotherapy with maximum decline after docetaxel treatment (*P* = 0.007). In addition, patients with lower residual cancer burden (0+I) and non-relapsing patients had higher serum miR-3200 levels. Importantly, miR-3200 expression was inversely correlated with lymph node clinical stage (r = -0.441, *P* = 0.021). Although functional studies are required to determine the mechanism of action for miR-3200, our results suggest that higher pre-treatment serum levels are associated with a better response and lower risk of relapse.

The oncogenic role of miR-21 and its anti-tumor-suppressive activities have been studied extensively in breast cancer; the miRNA levels positively correlate with increased resistance to chemotherapeutic drugs, such as doxorubicin and docetaxel [44]. In contrast to previous results, we found no significant alteration of serum miR-21 during chemotherapy treatment and no significant association with clinical outcome. However, other factors were correlated with miR-21 expression. In fact, miR-21 serum levels were significantly increased by the end of doxorubicin/cyclophosphamide treatment (point C) (*P* = 0.018). Previous studies have shown that only in TNBC is there a correlation between high miR-21 level and poor prognosis [45, 46]. The difference in findings might be due to the small number of triple-negative and Her2/*neu*-positive patients enrolled in our study.

## Conclusions

To our knowledge, this is the first report of miRNA profiling on a cohort of females with LABC undergoing NAC treatment. This study investigated multiple tissue and serum samples over time to describe the mechanistic changes occurring during treatment and to examine the use of miRNA expression as a biomarker. The use of miRNA as a biomarker is particularly intriguing, because it could replace the invasive method of tissue sampling with the relatively noninvasive method of blood sampling. Our model differs from previous studies; we used multiple samples taken at four time points, rather than pair-wise sampling. Expression of miR-3200 correlated with clinico-pathological feature, and elevated levels of serum miR-3200 were associated with better pathological response and a lower risk of relapse. However, the selection of this miRNA was predicted using a computational model and not previous literature; therefore, its function and mechanism of action remain largely unexplored. Our data propose for the first time the potential role of miR-3200 as a tumor-suppressive marker, though functional studies are still required to decipher its role in cancer.

## Supporting Information

S1 Table MicroRNA expression profiling of normal tissue versus tumor. (PDF)
