# Supplementary material for: Tissue and Serum miRNA Profile in Locally Advanced Breast Cancer (LABC) in Response to Neo-Adjuvant Chemotherapy (NAC) Treatment
Source: PLoS One. 2016 Apr 11;11(4):e0152032. doi: 10.1371/journal.pone.0152032 (PMC4827834; doi:10.1371/journal.pone.0152032)
Supplement: S1 Table — (PDF) [file pone.0152032.s001.pdf]

Supplementary Table 1 (S 1 Table): MicroRNA expression profiling of normal tissue versus tumor

Transcript ID	p-value(Tumor vs. Normal)	Fold-Change (Tumor vs. Normal)	Fold-Change (Tumor vs. Normal) (Description)
hsa-miR-141_st	8.69E-06	31.9879	Tumor up vs Normal
hsa-miR-203_st	1.23E-06	29.871	Tumor up vs Normal
hsa-miR-205_st	0.00015685	25.6819	Tumor up vs Normal
hsa-miR-375_st	0.0014072	21.619	Tumor up vs Normal
hsa-miR-183_st	0.000188633	21.3486	Tumor up vs Normal
hsa-miR-200a_st	0.00116584	19.5027	Tumor up vs Normal
hsa-miR-200b_st	0.00266865	15.609	Tumor up vs Normal
hsa-miR-196a_st	2.31E-05	14.0201	Tumor up vs Normal
hsa-miR-21_st	6.31E-06	12.3081	Tumor up vs Normal
hsa-miR-192_st	0.000220034	11.4991	Tumor up vs Normal
hsa-miR-200c_st	0.000449693	10.6507	Tumor up vs Normal
hsa-miR-1246_st	0.000122559	9.89497	Tumor up vs Normal
hsa-miR-148a_st	9.33E-05	8.62015	Tumor up vs Normal
hsa-miR-200b-star_st	0.00303628	7.82523	Tumor up vs Normal
hsa-miR-194_st	0.00112899	7.40861	Tumor up vs Normal
hsa-miR-200a-star_st	0.00098515	7.16065	Tumor up vs Normal
hsa-miR-181d_st	8.79E-05	6.90737	Tumor up vs Normal
hsa-miR-17-star_st	0.000235969	6.81062	Tumor up vs Normal
hsa-miR-182_st	0.000719531	6.70186	Tumor up vs Normal
hsa-miR-1244_st	0.00147737	6.55484	Tumor up vs Normal
hsa-miR-183-star_st	0.00264432	6.53105	Tumor up vs Normal
hsa-miR-21-star_st	1.26E-05	6.39487	Tumor up vs Normal
hsa-miR-339-3p_st	0.000219378	6.21964	Tumor up vs Normal
hsa-miR-1270_st	9.74E-05	6.03729	Tumor up vs Normal
hsa-miR-1290_st	0.00106542	5.71004	Tumor up vs Normal
hsa-miR-128_st	0.000534636	5.30256	Tumor up vs Normal
hsa-miR-27b-star_st	3.32E-06	5.11498	Tumor up vs Normal
hsa-miR-181a-2-star_st	3.33E-05	5.06199	Tumor up vs Normal
hsa-miR-148b_st	0.00118871	4.98992	Tumor up vs Normal
hsa-miR-30e_st	0.000221725	4.94081	Tumor up vs Normal
hsa-miR-1972_st	0.000106522	4.91524	Tumor up vs Normal
hsa-miR-331-5p_st	7.75E-05	4.69535	Tumor up vs Normal
hsa-miR-181c_st	0.000673685	4.58743	Tumor up vs Normal
hsa-miR-29c_st	0.000990462	4.57106	Tumor up vs Normal
hsa-miR-181c-star_st	0.000217651	4.30407	Tumor up vs Normal
hsa-miR-146b-5p_st	0.000733053	4.18683	Tumor up vs Normal
hsa-miR-769-3p_st	5.28E-05	4.15041	Tumor up vs Normal
hsa-miR-671-5p_st	0.00139497	3.96187	Tumor up vs Normal
hsa-miR-877_st	0.000276854	3.94234	Tumor up vs Normal
hsa-miR-19a_st	0.00148001	3.91864	Tumor up vs Normal

hsa-miR-3200_st	0.00217382	3.8768	Tumor up vs Normal
hsa-miR-200c-star_st	0.000954418	3.85953	Tumor up vs Normal
hsa-miR-505_st	0.00187586	3.69169	Tumor up vs Normal
hsa-miR-93-star_st	0.00167984	3.68391	Tumor up vs Normal
hsa-miR-34a-star_st	0.000944519	3.57025	Tumor up vs Normal
hsa-miR-4269_st	0.00131261	3.51862	Tumor up vs Normal
hsa-miR-130b_st	0.00160372	3.34963	Tumor up vs Normal
hsa-miR-125a-3p_st	0.000458697	3.26769	Tumor up vs Normal
hsa-miR-30b-star_st	0.000230343	3.13268	Tumor up vs Normal
hsa-miR-491-5p_st	0.00195261	3.12073	Tumor up vs Normal
hsa-miR-425_st	0.00229416	3.10943	Tumor up vs Normal
hsa-miR-195-star_st	0.00199726	3.07805	Tumor up vs Normal
hsa-miR-181a-star_st	0.00144829	2.87996	Tumor up vs Normal
hsa-miR-148a-star_st	0.00141841	2.8079	Tumor up vs Normal
hsa-miR-7-1-star_st	0.00163171	2.79416	Tumor up vs Normal
hsa-miR-331-3p_st	0.001159	2.72495	Tumor up vs Normal
hsa-miR-212_st	0.00178155	2.71532	Tumor up vs Normal
hsa-miR-188-5p_st	0.000460769	2.63863	Tumor up vs Normal
hp_hsa-mir-182_st	0.000101797	2.61251	Tumor up vs Normal
hsa-miR-23b-star_st	0.000697594	2.49684	Tumor up vs Normal
hsa-miR-19b_st	0.00159141	2.46944	Tumor up vs Normal
hp_hsa-mir-200c_st	7.18E-05	2.46309	Tumor up vs Normal
hsa-miR-146b-3p_st	0.000382052	2.4241	Tumor up vs Normal
hsa-miR-155_st	0.00318701	2.39375	Tumor up vs Normal
hsa-miR-138_st	0.00249203	2.39182	Tumor up vs Normal
hsa-miR-671-3p_st	0.00313614	2.39037	Tumor up vs Normal
hsa-miR-151-3p_st	0.000647978	2.32754	Tumor up vs Normal
hsa-miR-342-3p_st	0.00206295	2.31469	Tumor up vs Normal
hsa-miR-92b_st	0.00163438	2.28382	Tumor up vs Normal
hsa-miR-1201_st	0.000155723	2.27017	Tumor up vs Normal
hsa-miR-374b_st	0.00163283	2.15415	Tumor up vs Normal
hsa-miR-25_st	0.00262058	2.15116	Tumor up vs Normal
hsa-miR-34c-3p_st	0.00186057	2.09225	Tumor up vs Normal
hsa-miR-1306_st	0.00125234	2.00474	Tumor up vs Normal
hsa-miR-1207-5p_st	0.000516931	-2.11399	Tumor down vs Normal
hsa-miR-3196_st	0.00266106	-2.17586	Tumor down vs Normal
hsa-miR-762_st	0.00222946	-2.1809	Tumor down vs Normal
hsa-miR-145_st	0.00271042	-2.19978	Tumor down vs Normal
hsa-miR-886-5p_st	0.00105397	-2.37752	Tumor down vs Normal
hp_hsa-mir-3180-2_s_st	0.00161426	-2.38737	Tumor down vs Normal
hsa-miR-1469_st	0.00230837	-2.42673	Tumor down vs Normal
hsa-miR-638_st	0.00047486	-2.49726	Tumor down vs Normal
hsa-miR-4270_st	0.000255324	-2.53704	Tumor down vs Normal
hsa-miR-149-star_st	0.000454156	-2.59785	Tumor down vs Normal
hsa-miR-193a-5p_st	0.000230353	-2.62772	Tumor down vs Normal
hsa-miR-2861_st	0.000361638	-2.75822	Tumor down vs Normal

hsa-miR-1228_st	0.00122152	-2.77862	Tumor down vs Normal
hsa-miR-4281_st	0.000734223	-2.88867	Tumor down vs Normal
hsa-miR-1228-star_st	0.000263867	-2.95431	Tumor down vs Normal
hsa-miR-1281_st	0.000177245	-3.01656	Tumor down vs Normal
hsa-miR-1908_st	0.00031008	-3.10052	Tumor down vs Normal
hsa-miR-1268_st	5.61E-05	-3.12274	Tumor down vs Normal
hsa-miR-3141_st	3.93E-05	-3.31919	Tumor down vs Normal
hsa-miR-3185_st	0.000544416	-3.93214	Tumor down vs Normal
hsa-miR-1825_st	0.000289509	-4.13451	Tumor down vs Normal
hsa-miR-139-3p_st	0.00158872	-5.08063	Tumor down vs Normal
hsa-miR-139-5p_st	0.000126161	-5.83891	Tumor down vs Normal
hsa-miR-224-star_st	0.000701403	-6.63379	Tumor down vs Normal
hsa-miR-486-5p_st	0.00209272	-6.90209	Tumor down vs Normal
